# Role of Transjugular Intrahepatic Portosystemic Shunt in the Liver Transplant Setting

**DOI:** 10.3390/jcm13020600

**Published:** 2024-01-21

**Authors:** Simone Di Cola, Lucia Lapenna, Jakub Gazda, Stefano Fonte, Giulia Cusi, Samuele Esposito, Marco Mattana, Manuela Merli

**Affiliations:** 1 Department of Translational and Precision Medicine, Sapienza University of Rome, 00185 Rome, Italy; simone.dicola@uniroma1.it (S.D.C.); lucia.lapenna@uniroma1.it (L.L.); stefano.fonte@uniroma1.it (S.F.); giulia.cusi@uniroma1.it (G.C.); esposito.1891603@studenti.uniroma1.it (S.E.); marco.mattana@uniroma1.it (M.M.); 2 2nd Department of Internal Medicine, PJ Safarik University and L. Pasteur University Hospital in Kosice, 040 11 Kosice, Slovakia; jkgazda@gmail.com

**Keywords:** transjugular intrahepatic portosystemic shunt, TIPS, liver transplantation, bridge to transplant, timing

## Abstract

Liver transplantation is currently the only curative therapy for patients with liver cirrhosis. Not all patients in the natural course of the disease will undergo transplantation, but the majority of them will experience portal hypertension and its complications. In addition to medical and endoscopic therapy, a key role in managing these complications is played by the placement of a transjugular intrahepatic portosystemic shunt (TIPS). Some indications for TIPS placement are well-established, and they are expanding and broadening over time. This review aims to describe the role of TIPS in managing patients with liver cirrhosis, in light of liver transplantation. As far as it is known, TIPS placement seems not to affect the surgical aspects of liver transplantation, in terms of intraoperative bleeding rates, postoperative complications, or length of stay in the Intensive Care Unit. However, the placement of a TIPS “towards transplant” can offer advantages in terms of ameliorating a patient’s clinical condition at the time of transplantation and improving patient survival. Additionally, the TIPS procedure can help preserve the technical feasibility of the transplant itself. In this context, indications for TIPS placement at an earlier stage are drawing particular attention. However, TIPS insertion in decompensated patients can also lead to serious adverse events. For these reasons, further studies are needed to make reliable recommendations for TIPS in the pre-transplant setting.

## 1. Introduction

Cirrhosis represents an advanced stage of chronic liver disease, characterized by an iz okunfavorable prognosis and associated with 2.4% of global deaths in 2019 [1] due to liver decompensation. Complications of liver cirrhosis arise from liver failure and the development of portal hypertension leading to a progressive worsening of the disease. The mortality rate ranges from less than 1% per year in patients with compensated cirrhosis to more than 20% per year after the first decompensation [2,3].

**Figure 1:**
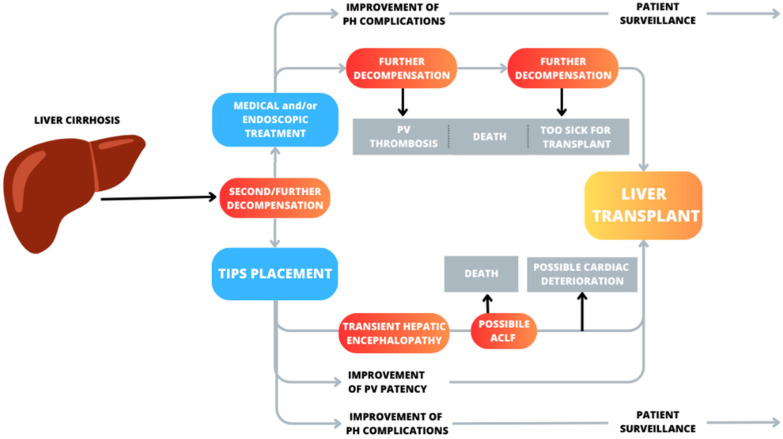
Possible scenarios after second or further decompensation in patients with liver cirrhosis treated with medical therapy versus TIPS placement with a view to transplantation. Red boxes indicate possible negative events and the progression of liver diseases. Grey boxes indicate potential causes of a patient’s exclusion from transplantation or death. Patients with decompensated liver cirrhosis undergoing medical or endoscopic therapy may experience clinical improvement, but the natural history of the disease may progress to further deterioration and a need for transplantation. Patients with TIPS can often develop hepatic encephalopathy, which is frequently transient, and seldom ACLF or cardiac deterioration. Some patients after a TIPS may correct portal thrombosis, making liver transplants more feasible. TIPS, by improving portal hypertension-related complications, may increase transplant-free survival. HE, hepatic encephalopathy; TIPS, transjugular intrahepatic portosystemic shunt, PH, portal hypertension; ACLF, acute-on-chronic liver failure; PV, portal vein.

To treat complications of portal hypertension, chronic medical treatment is indicated, with diuretics and nonselective beta-blockers (NSBBs) [4,5] or endoscopic band ligation. However, during the natural history of cirrhosis, some patients may develop difficult-to-treat/refractory ascites or a high risk of recurrent gastrointestinal (GI) bleeding, requiring a transjugular intrahepatic portosystemic shunt (TIPS) procedure [6]. Following TIPS placement, the pressure in the portal venous system drops, reducing the risk of variceal bleeding and improving ascites. Due to the treatment of portal hypertension (PH), a TIPS may even improve patient survival [7], but the progression of liver failure and its consequences frequently leave liver transplant as the only definitive treatment in these patients. (Figure 1). For these reasons [8], during the clinical course of advanced liver disease, a TIPS may also be an opportunity to allow more patients to reach the time of liver transplant more safely.

## 2. TIPS toward Liver Transplant: Role and Timing

Liver transplantation represents the only curative treatment for patients with liver cirrhosis. Bridges to transplantation are used to manage patients who are listed for an organ transplant, acting to temporize whilst patients are on the waiting list. Various approaches can be chosen depending on the underlying medical condition and individual characteristics.

In patients awaiting liver transplant, TIPS placement can be an option to manage complications associated with portal hypertension, in order to reduce morbidity and mortality. Ascites, hydrothorax, and GI bleeding represent frequent and widely recognized indications for TIPS placement, based on the well-established literature data assessing complication management and mortality as outcomes [9,10]. However, a transplant perspective may anticipate the TIPS procedure to improve a patient’s clinical condition at the time of transplantation. Furthermore, in the case of portal vein thrombosis, the TIPS procedure could preserve the technical feasibility of the transplant itself. 

**Figure 2:**
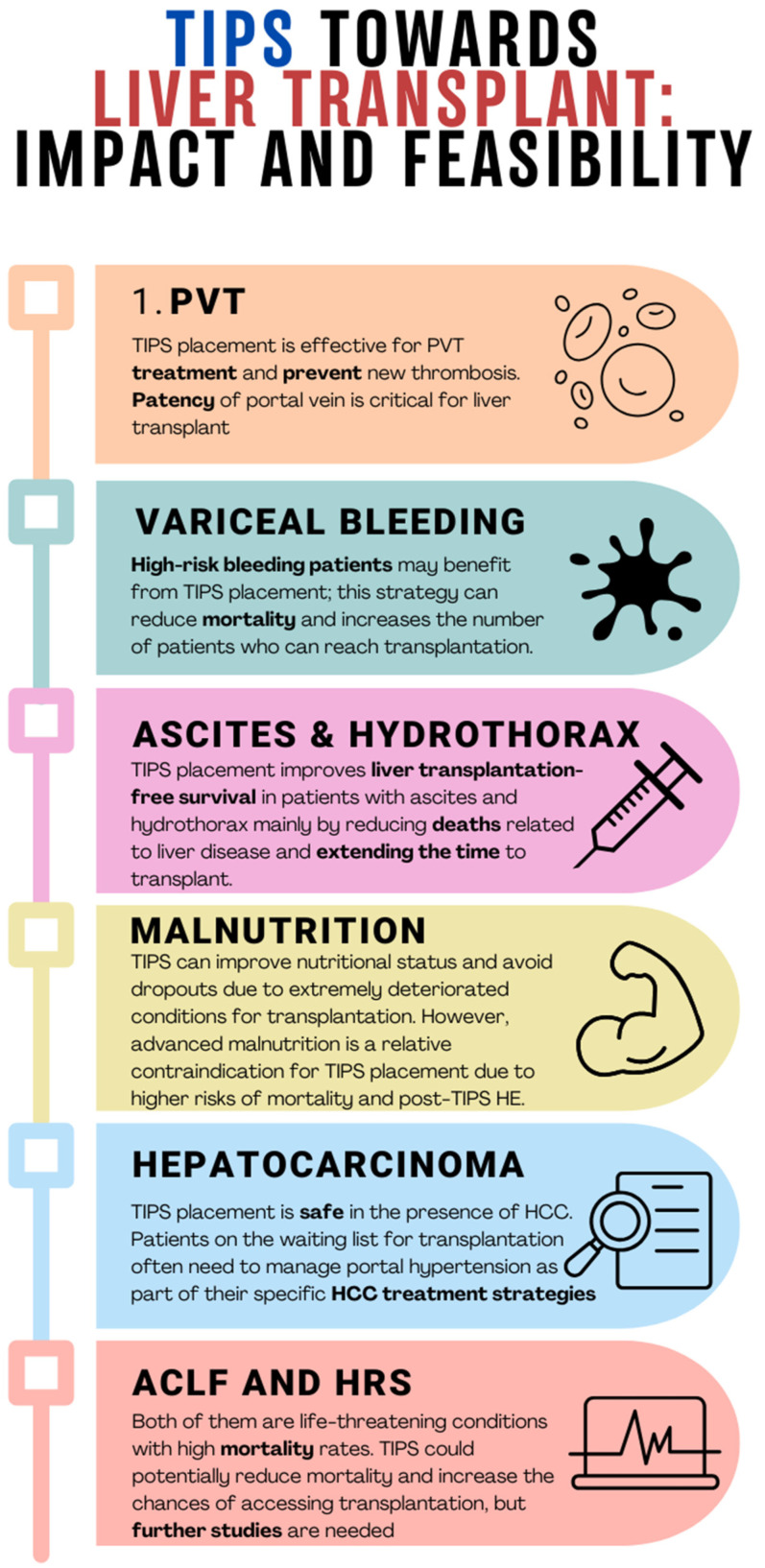
Clinical conditions that may occur in patients eligible for liver transplantation: impact and feasibility of TIPS. PVT, portal vein thrombosis; HCC, hepatocarcinoma; ACLF, acute-on-chronic liver failure; HRS, hepatorenal syndrome; HE, hepatic encephalopathy.

To better understand this concept, in this review, we aim to explore some liver transplantation settings, highlighting the possible impact and feasibility of TIPS (Figure 2). 

As it is known, TIPS is sometimes needed to treat complications of portal hypertension in a bridge-to-transplant setting, even in patients at high risk of decompensation and mortality. A recent meta-analysis based on individual data showed that, compared with standard therapy, the use of TIPS reduces the incidence of further decompensation events, regardless of the indication, and so, it increases survival in highly selected patients with variceal bleeding and refractory ascites [7]. Patients on a liver transplant waiting list are mostly individuals with end-stage liver disease, who frequently have already experienced multiple episodes of liver decompensation. While waiting for a liver graft, they may experience further decompensation and die before being transplanted. As recently demonstrated in a further meta-analysis and a previous study on the clinical course of cirrhosis [11], most deaths occur when episodes of decompensation become recurrent (Figure 1).

According to a French study published in 2022 [12], the dropout rate due to death or disease worsening in a list of 15,584 patients waiting for liver transplantation, during a 10-year observation time, was approximately 22%. Overall, 46.6% of these patients were listed for HCC. Similar results were obtained from a study on portal vein thrombosis (PVT) [13], showing dropout rates even greater than 20%, considering that PVT may also compromise the technical feasibility of liver transplantation. 

Indeed, PVT is a common event in cirrhosis, with a prevalence ranging from 2% to 26% in liver transplant candidates [14], and is associated with a 62% increase in the risk of mortality compared with cirrhotic patients without PVT [15]. PVT is also associated with increased mortality in the post-transplant period [16]. A large study conducted in a single center, involving over 3200 liver transplant candidates, found that those undergoing transplantation with an occlusive PVT had a sevenfold higher risk of mortality at 30 days compared with those without PVT [17]. One retrospective study using the Organ Procurement and Transplant Network (OPTN) database reported that the prevalence of PVT at candidate registration increased between 2002 and 2014 and that the presence of PVT was associated with an increased waitlist dropout [13].

TIPS placement can be difficult in the setting of PVT or cavernomatosis, even though recent studies suggest that the success rate of the procedure is improving due to new technical approaches [18,19]. In a recent randomized controlled study, the success rate of TIPS placement was very high and, in addition to excellent rebleeding control, TIPS also improved recanalization of the portal vein [20]. Anticoagulation (AC) represents the first-line therapeutic strategy for PTV. In a systematic review [21] and a recent prospective observational study [22], the efficacy of AC and TIPS placement in PVT were compared. Both strategies were found to be effective in achieving portal vein recanalization. 

However, anticoagulation therapy takes some time to act and can be difficult to perform in patients with advanced liver disease and very low platelet count. 

In a large systematic review, including 399 PVT patients (92% of whom had cirrhosis), the success rate of the TIPS procedure was approximately 95% (95% confidence interval, CI: 89–98%), even though patients were significantly heterogeneous, primarily due to a proportion of patients with cavernomatosis [23].

To face technical difficulties caused by a complete portal thrombosis or a portal cavernomatosis, alternative techniques have been described, such as the transhepatic and trans-splenic approaches, reaching almost 100% technical feasibility and allowing many patients previously excluded from transplantation to access it [18,19]. In our clinical practice, TIPS should be proposed when anticoagulant treatment is contraindicated or unsuccessful and the radiological approach is technically feasible, or in patients with transplant perspectives presenting with other complications of portal hypertension that would benefit from shunt placement. Indeed, portal vein recanalization and TIPS may improve a patient’s candidacy for liver transplantation [24]. 

Variceal bleeding represents one of the worst decompensations of liver cirrhosis, with a high rate of mortality. Endoscopic band ligation (EBL) and non-selective beta-blockers (NSBBs) represent the first step in the management of variceal bleeding, both in primary and secondary prophylaxis. However, there is an estimated 15–21% risk of treatment failure or rebleeding in these patients, causing a rate of mortality as high as 80% [25]. In this perspective, TIPS placement in high-risk variceal bleeding patients (pre-emptive TIPS) has been clearly demonstrated to be superior to the standard medical treatment in improving survival [26,27,28,29]. A recent individual patient meta-analysis showed that preemptive TIPS significantly increased the proportion of high-risk patients with cirrhosis and acute variceal bleeding who survived for 1 year, compared with drugs plus endoscopy (hazard ratio (HR) 0.443; 95% CI 0.323–0.607; *p* < 0.001) [30]. The placement of TIPS on time, reducing mortality and rapid deterioration of liver disease, allows more patients to access liver transplantation.

Refractory ascites is a common complication of advanced cirrhosis, involving 5–10% of patients [31]. Diuretics and repeated large volume paracentesis (LVP) are the first line strategy to manage ascites decompensation. However, as time passes, patients may experience a range of related complications, such as hypotension, acute kidney injury, hepatorenal syndrome, and spontaneous bacterial peritonitis.

TIPS placement is an effective therapy for refractory ascites and has been recently proposed also for recurrent ascites, even if results for survival benefits are controversial [32,33,34]. One meta-analysis analyzed aggregated individual patient data from four randomized controlled trials, revealing that TIPS led to a significant improvement in liver transplant-free (LTF) survival [35]. In another study, the proportions of liver disease-related deaths were 30% and 40% in the TIPS and paracentesis groups, respectively [36], and no significant difference was observed in the number of patients who underwent liver transplantation. Based on the presented data, it is apparent that two distinct patient cohorts (LVP versus TIPS) demonstrated comparable mortality rates but diverged significantly in terms of survival duration. The improved LTF observed in TIPS patients is primarily attributed to a reduction in portal hypertension-related mortality. Another contributing factor to enhanced LTF survival is the prolonged interval before liver transplantation, as demonstrated by two studies [37,38].

In a retrospective study, cirrhotic patients who underwent TIPS for refractory ascites were compared to similar patients who underwent serial paracentesis. After adjusting for patient characteristics, TIPS patients showed improved survival compared with those who underwent paracentesis, with significant differences in survival rates at 1, 2, and 3 years [39]. However, the patient eligible for TIPS should be carefully selected, taking into account the stage of liver disease, which serves as a warning for the high rate of decompensation and death after the procedure, although a retrospective study from 2019 showed that mortality in this kind of patient (MELD > 18) appears to be independent of the therapeutic strategy (LVP vs. TIPS) [40].

Malnutrition is a common feature of cirrhosis and significantly impacts the disease’s prognosis [41,42,43,44]. The rate of sarcopenia is directly related to the stage of liver disease [45]; for this reason, alterations in nutritional status can significantly impact the prognosis in patients waiting for liver transplantation [46]. Sarcopenia can sometimes represent a relative contraindication for surgery due to the higher rate of death or complication during and after liver transplantation [45,47].

A strong interrelation has been reported between TIPS placement and patient nutritional status. On the one hand, patients with advanced sarcopenia have a worse prognosis after TIPS in terms of hepatic decompensation, acute-on-chronic liver failure, and survival [48,49,50]. On the other hand, TIPS placement may improve nutritional status and sometimes can even reverse the condition of sarcopenia [51]. Montomoli et al., in a prospective observational study, investigated the effect of TIPS on malnutrition in portal hypertensive cirrhotic patients, and they observed that dry lean mass increased in low- or normal-weight patients after TIPS placement but did not in overweight patients [52]. It is still under debate whether TIPS placement could be anticipated in some patients with complications of portal hypertension and also to prevent advanced malnutrition and sarcopenia [51].

In this context, TIPS placement within a “therapeutic window” may lead to improved nutritional status for the patient and prevent advanced malnutrition from also becoming a risk factor for liver transplant surgery.

Lastly, it is important to note that cirrhosis is a chronic condition characterized by persistent and recurring complications, which significantly contribute to the overall burden of healthcare costs. In this context, TIPS, allowing a reduction in the rate of liver decompensation, a decrease in portal hypertension-related complications, and limiting frequent hospitalizations may lead to cost reduction, better management, and an improved economic impact [53]. This consideration could be particularly true if we consider patients with refractory/recurrent ascites who require frequent LVP, access to day hospitals or hospitalizations, and treatment for further decompensations. This savings would allow for a larger amount of resources to be available for diagnostic and therapeutic needs for patients awaiting liver transplantation.

The choice of the best timing for TIPS placement during the natural history of cirrhosis is at present an important topic with no definitive answers. However, even if we cannot provide, based on the current literature, a specific recommendation regarding the timing of TIPS placement, we suggest carefully considering and balancing indications and contraindications of the procedure at any new clinical event in order to manage portal hypertension complications and timely predict potentially unfavorable conditions for LT. In some patients, a multidisciplinary discussion and a team decision could be helpful to take care, accurately, of the complete patient’s individual context. Indeed, it is well-known that TIPS placement can also give rise to several adverse effects, including an acceleration of liver failure due to decreased blood supply [54,55], the exacerbation of cardiomyopathy [56,57], and the occurrence of HE [58,59], which stands out as the most prevalent clinical complication during the early period following TIPS placement. All these adverse events need to be taken carefully into account before the TIPS procedure. Nonetheless, recent evidence indicates that HE is preventable [60] and treatable in the majority of patients and does not impact a patient’s long-term prognosis [61].

Interestingly, a retrospective, single-center study purposely investigated the role of TIPS as a bridge therapy in liver transplant-eligible patients [62]. Of the 98 patients who were enrolled, 73 had TIPS placed before being listed, and 25 underwent the TIPS procedure while on the waiting list. Both groups were compared to a control group of 60 patients without TIPS. The timing of TIPS placement, before or after being listed, did not impact the survival rate before transplantation. The mortality rate of the patients on the waiting list was reported to have decreased after TIPS placement was introduced as an option, according to clinical indications compared with historical data reported in the same center. Some patients were even delisted due to clinical improvement after TIPS placement; in this latter group, the authors showed a similar 5-year overall survival rate compared with those who underwent liver transplantation.

In the past, TIPS was investigated as a potential technique to mitigate intraoperative blood loss during transplantation, thereby positively influencing short-term outcomes [63,64,65,66]. Moreno et al. conducted an extensive comparative longitudinal retrospective study of 875 patients aimed at assessing the short- and long-term outcomes of liver transplantation by comparing individuals with TIPS and those without. The study evaluated various endpoints, including the duration of surgery, cold ischemia time, warm ischemia time, blood product need, postoperative complications (both vascular and non-vascular), length of stay in the Intensive Care Unit (ICU), total hospital stay, re-transplantation rates, and 1- and 3-year survival rates. Remarkably, no statistically significant differences were observed between the two groups in any of these parameters [67]. Similar conclusions were reported by Dell’Era et al., who found no significant differences between patients with prior TIPS placement and a control group with regard to transfusion requirements, operative time, overall length of hospital stay, ICU length of stay, and complication rates [68].

A subsequent prospective study conducted on a large sample of 591 patients confirmed that the presence of TIPS had no influence on intra-operative parameters or post-transplant survival up to 5 years [69]. Comparing the largest studies on the subject, Barbieri et al. concluded that the placement of TIPS before transplantation had no significant impact on either perioperative parameters or survival at 1 month and 1, 3, and 5 years after liver transplant [70]. 

It is important to highlight that the TIPS procedure also has well-known contraindications such as significant pulmonary hypertension, heart failure or severe cardiac valvular insufficiency, severe liver failure, chronic hepatic encephalopathy not controlled with medical therapy, and sepsis [10,71,72]. For this reason, an accurate patient selection is crucial. This holds true in general, but it becomes even more important in the context of liver transplantation. Indeed, in the latest AASLD TIPS guidelines [24] and similarly, in the Baveno guidelines [9] for variceal bleeding, patients with a MELD score > 30, lactate > 12 mmol/L, or Child–Pugh > 13, salvage/rescue TIPS should not be used unless TIPS is a bridge to liver transplantation in the short term. This highlights how patient selection is fundamental and sometimes requires a case-by-case evaluation.

In conclusion, TIPS placement is effective in managing complications associated with portal hypertension in cirrhotic patients and may serve as an excellent tool to potentially prevent deaths while on the waiting list for liver transplantation. The TIPS procedure could also play a specific role in restoring portal vein patency in patients under consideration for liver transplant in whom portal thrombosis occurs. Due to the complexity of the decision and the need to consider a number of relevant issues when positioning a TIPS in a patient with advanced liver disease waiting for liver transplantation, a multidisciplinary team decision is recommended. There is no impact of having a prior TIPS during the surgical procedure of liver transplantation on the duration of surgery, blood product need, postoperative complications, length of stay in the Intensive Care Unit, total hospital stay, or re-transplantation rates.

## 3. TIPS in “Special” Liver Transplant Settings

### 3.1. Hepatocellular Carcinoma (HCC)

TIPS placement in patients with clinically significant portal hypertension and HCC has proven to be feasible and effective in selected cases [73,74].

The major safety issues are related to the size of the tumor, the possibility that the nodule location interferes with the trajectory of the stent, and the eventual risk of distant metastasis caused by the procedure [75]. Definite answers about these issues are lacking and, in the majority of cases, the decision will need a team discussion between the radiologist, the hepatologist, and the surgeon. 

Between the late twentieth century and the early 2000s, some studies suggested that the TIPS procedure could somehow increase the risk of HCC in cirrhotic patients [76,77]. This finding was supported thanks to the hypothesis that TIPS could lead to vascular alterations, resulting in hypoxia triggers and therefore the development of hepatocellular carcinoma. Further studies, also included in a recent meta-analysis, failed to demonstrate any association between the presence of TIPS and the onset of an HCC (risk ratio: 1.37, (CI): 0.96–1.97; *p* = 0.08) [78].

Other studies have investigated the safety of transarterial chemoembolization (TACE), radiofrequency ablation (RFTA), and radioembolization in patients with TIPS [79,80,81,82,83]. These locoregional treatments were feasible in patients with TIPS, but they were utilized only in selected patients with adequate liver function. Indeed, other studies evidenced an increased risk of hepatotoxicity and hepatic encephalopathy [84] with this combined approach. In addition, Kuo et al. demonstrated that, although feasible, TACE in patients bearing TIPS is associated with worse outcomes than in non-TIPS patients, both in terms of safety and efficacy [85]. Nevertheless, the 3-year overall survival did not reveal significant differences. 

In conclusion, locoregional treatment for hepatocellular carcinoma, which is a frequent need in patients on a waiting list for liver transplantation, is feasible in patients with TIPS but may further deteriorate liver function. Multidisciplinary decisions with the radiologist, the hepatologist, and liver transplant surgeons are recommended.

### 3.2. Acute-on-Chronic Liver Failure (ACLF) 

Acute-on-chronic liver failure (ACLF) is a subtype of acutely decompensated cirrhosis, which is associated with a significantly higher 28-day mortality rate when compared with acutely decompensated cirrhosis without ACLF (more than 20% versus less than 5%), as reported in a study published in *Gastroenterology* [86]. Currently, there is a dearth of studies assessing the impact of TIPS insertion on the disease course when ACLF itself is the indication for TIPS placement. It is worth noting that, despite having a plausible pathophysiological rationale (acting upstream on the pathophysiological pathway—portal hypertension), TIPS insertion is often infeasible in most ACLF patients due to kidney and brain dysfunction. Due to the lack of conclusive evidence supporting TIPS placement in ACLF patients alone, it cannot be recommended as a bridge-to-transplant therapy.

Additionally, two distinct scenarios arise concerning ACLF and variceal bleeding. The first scenario is variceal bleeding as a precipitating factor of ACLF, and the second is variceal bleeding as a complication of ACLF. The management of variceal bleeding outside the context of ACLF is generally accepted and includes consideration of pre-emptive or salvage TIPS procedures [9,87]. The management of patients with variceal bleeding as a complication of ACLF is similar to that of patients without ACLF.

Numerous studies have explored both preemptive and salvage TIPS placements. Gu et al. recently published a comprehensive narrative review, highlighting that preemptive TIPS benefits ACLF patients when compared with non-ACLF patients and that salvage TIPS improves one-year mortality rates in patients with ACLF grade 1, albeit not in grades 2 or 3 [88]. Based on this emerging evidence, preemptive TIPS may be taken into consideration as a bridge-to-transplant option in cirrhotic patients with ACLF and variceal bleeding.

### 3.3. Hepatorenal Syndrome

Hepatorenal syndrome (HRS) is a condition characterized by kidney dysfunction in cirrhotic patients, primarily attributed to a reduction in renal blood flow despite the kidneys being histologically normal. HRS can be further classified into two categories: “acute kidney injury” (HRS-AKI, formerly known as HRS Type I) and “non-acute kidney injury” (HRS-NAKI, formerly known as HRS Type II). The conventional approach to managing HRS-AKI involves discontinuing diuretic use and administering albumin infusions in conjunction with vasoactive agents, such as terlipressin. Patients who do not respond to this treatment should be considered for liver transplantation, similar to HRS-NAKI patients.

Numerous retrospective observational studies have indicated that TIPS can ameliorate kidney function. Wong et al. reported that in HRS-AKI patients, TIPS not only initially improved kidney function but also led to the normalization of plasma renin and aldosterone levels, glomerular filtration rate, and urinary sodium excretion 12 months after the procedure [89]. Similarly, Testino et al. demonstrated significant improvements in serum creatinine and blood urea nitrogen 30 days after TIPS insertion in patients with alcoholic hepatitis and HRS-1 (historically known as HRS-AKI) [90]. However, both studies may be affected by small sample sizes and lack comparisons to cohorts receiving a standard treatment.

Brensing et al. observed improved kidney function, as evidenced by increased creatinine clearance and sodium excretion, within two weeks following TIPS in patients with HRS (both Type I and II) compared with a control group. They also noted enhanced 3-, 6-, 12-, and 18-month survival rates in TIPS patients [91]. Nevertheless, a recent meta-analysis revealed a favorable trend in short-term and one-year survival among HRS patients treated with TIPS. However, the authors acknowledged limitations, including small sample sizes and potential patient selection bias in the underlying studies [92]. Consequently, further investigation is needed before recommending TIPS as a bridge-to-transplant option for any type of HRS patient.

Considering this need for additional research, it is worth noting that ongoing work is already underway. Ripoll et al. recently published a study protocol investigating whether TIPS is superior to the standard of care in patients with HRS-AKI [93].

### 3.4. Cirrhotic Cardiomyopathy

Cirrhotic cardiomyopathy (CCM) is a distinct clinical syndrome, characterized by systolic and/or diastolic dysfunction resulting from peripheral vasodilation, complicated by hemodynamic derangement and prolonged activation of compensatory mechanisms. However, the pathophysiology is far more complex and beyond the scope of this article. CCM has been reported in 30–70% of patients with cirrhosis [94] and may become clinically evident in conditions of rapid and significant heart overload such as in the first weeks after TIPS placement [95].

Some studies evaluated the short- and medium-term occurrence of cardiac decompensation and mortality following TIPS in patients with previously documented cirrhotic cardiomyopathy. Radunski et al. observed a significant increase in cardiac chamber volumes following TIPS; however, after a median follow-up time of 207 days, no patients experienced cardiac decompensation or heart-related death [96]. Fili et al. followed 15 patients with decompensated cirrhosis (33% with diastolic dysfunction) and did not observe any change in cardiac function or any episode of cardiac decompensation one month after TIPS placement [97]. On the other hand, Meucci et al. followed a mixed cohort of patients (with and without CCM) for a median of 36 months after TIPS and found a higher all-cause mortality rate in patients with higher grades of diastolic dysfunction before the TIPS procedure [98]. Similarly, diastolic dysfunction, indicated by a reduced E/A ratio, is associated with reduced ascites clearance and increased mortality post-TIPS [99]. 

Following these observations, and also due to the scarcity of data in the setting of TIPS as a bridge-to-transplant, caution should be taken with regard to TIPS positioning shortly before liver transplant. Even in patients with otherwise compensated CCM, the TIPS procedure may trigger symptoms of cardiac overload in the short term, therefore suggesting a time lag for cardiac monitoring before the surgery takes place. Indeed, current evidence suggests an initial risk of cardiac decompensation following liver transplantation, although cardiac function improvement, resulting from a reversal of hyperdynamic circulation 6–12 months after liver transplantation, has been reported [94].

## 4. Conclusions

TIPS placement in patients with liver cirrhosis is becoming increasingly common in addressing both acute and chronic complications of portal hypertension. The decision to place TIPS in a patient potentially eligible for a liver transplant should be carefully undertaken. This procedure may be life-saving in some conditions (GI bleeding) or may even relieve some complications (refractory ascites), facilitating “the patient’s journey” to liver transplantation. At the same time, these patients, due to advanced liver disease, may manifest more severe consequences. Therefore, the timing of TIPS placement within a “therapeutic window” is crucial to maximize benefits without causing adverse events. In the context of liver transplantation, TIPS could potentially prevent certain liver complications that serve as contraindications to transplantation itself, such as advanced malnutrition or complete portal vein thrombosis.

There are currently insufficient studies assessing the impact of TIPS on waitlist mortality and post-transplant survival, and such studies will be necessary in the near future to reach evidence-based recommendations.

However, what we can state regarding the management of portal hypertension complications is that TIPS plays a crucial role with indications that are increasing and expanding. Therefore, patients should be evaluated from this point of view, especially if they are young and have a transplant perspective, with a case-by-case and multidisciplinary approach. 

## References

[1] https://www.who.int/data/gho/data/themes/mortality-and-global-health-estimates/ghe-leading-causes-of-death.

[2] D’Amico G., Garcia-Tsao G., Pagliaro L. (2006). Natural history and prognostic indicators of survival in cirrhosis: A systematic review of 118 studies. J. Hepatol..

[3] D’Amico G., Morabito A., D’Amico M., Pasta L., Malizia G., Rebora P., Valsecchi M.G. (2018). Clinical states of cirrhosis and competing risks. J. Hepatol..

[4] Rudler M., Mallet M., Sultanik P., Bouzbib C., Thabut D. (2020). Optimal management of ascites. Liver Int..

[5] Jakab S.S., Garcia-Tsao G. (2020). Evaluation and Management of Esophageal and Gastric Varices in Patients with Cirrhosis. Clin. Liver Dis..

[6] Smith M., Durham J. (2016). Evolving Indications for Tips. Tech. Vasc. Interv. Radiol..

[7] Larrue H., D’Amico G., Olivas P., Lv Y., Bucsics T., Rudler M., Sauerbruch T., Hernandez-Gea V., Han G., Reiberger T. (2023). TIPS prevents further decompensation and improves survival in patients with cirrhosis and portal hypertension in an individual patient data meta-analysis. J. Hepatol..

[8] Bodzin A.S., Baker T.B. (2018). Liver Transplantation Today: Where We Are Now and Where We Are Going. Liver Transplant..

[9] De Franchis R., Bosch J., Garcia-Tsao G., Reiberger T., Ripoll C., Baveno VII Faculty (2022). Baveno VII—Renewing consensus in portal hypertension. J. Hepatol..

[10] Tripathi D., Stanley A.J., Hayes P.C., Travis S., Armstrong M.J., Tsochatzis E.A., Rowe I.A., Roslund N., Ireland H., Lomax M. (2020). Transjugular intrahepatic portosystemic stent-shunt in the management of portal hypertension. Gut.

[11] Villanueva C., Torres F., Sarin S.K., Shah H.A., Tripathi D., Brujats A., Rodrigues S.G., Bhardwaj A., Azam Z., Hayes P.C. (2022). Carvedilol reduces the risk of decompensation and mortality in patients with compensated cirrhosis in a competing-risk meta-analysis. J. Hepatol..

[12] Delacôte C., Favre M., El Amrani M., Ningarhari M., Lemaitre E., Ntandja-Wandji L.C., Bauvin P., Boleslawski E., Millet G., Truant S. (2022). Morbid obesity increases death and dropout from the liver transplantation waiting list: A prospective cohort study. United Eur. Gastroenterol. J..

[13] Montenovo M., Rahnemai-Azar A., Reyes J., Perkins J. (2018). Clinical Impact and Risk Factors of Portal Vein Thrombosis for Patients on Wait List for Liver Transplant. Exp. Clin. Transplant..

[14] Ponziani F.R., Zocco M.A., Senzolo M., Pompili M., Gasbarrini A., Avolio A.W. (2014). Portal vein thrombosis and liver transplantation: Implications for waiting list period, surgical approach, early and late follow-up. Transplant. Rev..

[15] Stine J.G., Shah P.M., Cornella S.L., Rudnick S.R., Ghabril M.S., Stukenborg G.J., Northup P.G. (2015). Portal vein thrombosis, mortality and hepatic decompensation in patients with cirrhosis: A meta-analysis. World J. Hepatol..

[16] Ghabril M., Agarwal S., Lacerda M., Chalasani N., Kwo P., Tector A.J. (2016). Portal Vein Thrombosis Is a Risk Factor for Poor Early Outcomes after Liver Transplantation: Analysis of Risk Factors and Outcomes for Portal Vein Thrombosis in Waitlisted Patients. Transplantation.

[17] Englesbe M.J., Kubus J., Muhammad W., Sonnenday C.J., Welling T., Punch J.D., Lynch R.J., Marrero J.A., Pelletier S.J. (2010). Portal vein thrombosis and survival in patients with cirrhosis. Liver Transpl..

[18] Thornburg B., Desai K., Hickey R., Kulik L., Ganger D., Baker T., Abecassis M., Lewandowski R.J., Salem R. (2016). Portal Vein Recanalization and Transjugular Intrahepatic Portosystemic Shunt Creation for Chronic Portal Vein Thrombosis: Technical Considerations. Tech. Vasc. Interv. Radiol..

[19] Salem R., Vouche M., Baker T., Herrero J.I., Caicedo J.C., Fryer J., Hickey R., Habib A., Abecassis M., Koller F. (2015). Pretransplant Portal Vein Recanalization-Transjugular Intrahepatic Portosystemic Shunt in Patients with Complete Obliterative Portal Vein Thrombosis. Transplantation.

[20] Lv Y., Qi X., He C., Wang Z., Yin Z., Niu J., Guo W., Bai W., Zhang H., Xie H. (2018). Covered TIPS versus endoscopic band ligation plus propranolol for the prevention of variceal rebleeding in cirrhotic patients with portal vein thrombosis: A randomised controlled trial. Gut.

[21] Davis J.P.E., Ogurick A.G., Rothermel C.E., Sohn M.W., Intagliata N.M., Northup P.G. (2019). Anticoagulation and Transjugular Intrahepatic Portosystemic Shunting for Treatment of Portal Vein Thrombosis in Cirrhosis: A Systematic Review and Meta-Analysis. Clin. Appl. Thromb./Hemost..

[22] Lv Y., Bai W., Li K., Wang Z., Guo W., Luo B., Wang J., Wang Q., Wang E., Xia D. (2022). Anticoagulation and Transjugular Intrahepatic Portosystemic Shunt for the Management of Portal Vein Thrombosis in Cirrhosis: A Prospective Observational Study. Am. J. Gastroenterol..

[23] Rodrigues S.G., Sixt S., Abraldes J.G., De Gottardi A., Klinger C., Bosch J., Baumgartner I., Berzigotti A. (2019). Systematic review with meta-analysis: Portal vein recanalisation and transjugular intrahepatic portosystemic shunt for portal vein thrombosis. Aliment. Pharmacol. Ther..

[24] Lee E.W., Eghtesad B., Garcia-Tsao G., Haskal Z.J., Hernandez-Gea V., Jalaeian H., Kalva S.P., Mohanty A., Thabut D., Abraldes J.G. (2024). AASLD Practice Guidance on the use of TIPS, variceal embolization, and retrograde transvenous obliteration in the management of variceal hemorrhage. Hepatology.

[25] Asrani S.K., Kamath P.S. (2013). Natural history of cirrhosis. Curr. Gastroenterol. Rep..

[26] Monescillo A., Martínez-Lagares F., Ruiz-del-Arbol L., Sierra A., Guevara C., Jiménez E., Marrero J.M., Buceta E., Sánchez J., Castellot A. (2004). Influence of portal hypertension and its early decompression by TIPS placement on the outcome of variceal bleeding. Hepatology.

[27] García-Pagán J.C., Caca K., Bureau C., Laleman W., Appenrodt B., Luca A., Abraldes J.G., Nevens F., Vinel J.P., Mössner J. (2010). Early use of TIPS in patients with cirrhosis and variceal bleeding. N. Engl. J. Med..

[28] Manning C., Elzubeir A., Alam S. (2021). The role of pre-emptive Transjugular Intrahepatic Portosystemic Shunt in acute variceal bleeding: A literature review. Ther. Adv. Chronic Dis..

[29] Hussain I., Wong Y.J., Lohan R., Lin S., Kumar R. (2022). Does preemptive transjugular intrahepatic portosystemic shunt improve survival after acute variceal bleeding? Systematic review, meta-analysis, and trial sequential analysis of randomized trials. J. Gastroenterol. Hepatol..

[30] Nicoară-Farcău O., Han G., Rudler M., Angrisani D., Monescillo A., Torres F., Casanovas G., Bosch J., Lv Y., Thabut D. (2021). Effects of Early Placement of Transjugular Portosystemic Shunts in Patients with High-Risk Acute Variceal Bleeding: A Meta-analysis of Individual Patient Data. Gastroenterology.

[31] Arroyo V., Colmenero J. (2003). Ascites and hepatorenal syndrome in cirrhosis: Pathophysiological basis of therapy and current management. J. Hepatol..

[32] Ginès P., Uriz J., Calahorra B., Garcia-Tsao G., Kamath P.S., Del Arbol L.R., Planas R., Bosch J., Arroyo V., Rodés J. (2002). Transjugular intrahepatic portosystemic shunting versus paracentesis plus albumin for refractory ascites in cirrhosis. Gastroenterology.

[33] Narahara Y., Kanazawa H., Fukuda T., Matsushita Y., Harimoto H., Kidokoro H., Katakura T., Atsukawa M., Taki Y., Kimura Y. (2011). Transjugular intrahepatic portosystemic shunt versus paracentesis plus albumin in patients with refractory ascites who have good hepatic and renal function: A prospective randomized trial. J. Gastroenterol..

[34] D’Amico G., Luca A., Morabito A., Miraglia R., D’Amico M. (2005). Uncovered transjugular intrahepatic portosystemic shunt for refractory ascites: A meta-analysis. Gastroenterology.

[35] Salerno F., Cammà C., Enea M., Rössle M., Wong F. (2007). Transjugular intrahepatic portosystemic shunt for refractory ascites: A meta-analysis of individual patient data. Gastroenterology.

[36] Bai M., Qi X.S., Yang Z.P., Yang M., Fan D.M., Han G.H. (2014). TIPS improves liver transplantation-free survival in cirrhotic patients with refractory ascites: An updated meta-analysis. World J. Gastroenterol..

[37] Sanyal A.J., Genning C., Reddy K.R., Wong F., Kowdley K.V., Benner K., McCashland T., North American Study for the Treatment of Refractory Ascites Group (2003). The North American Study for the Treatment of Refractory Ascites. Gastroenterology.

[38] Salerno F., Cazzaniga M., Pagnozzi G., Cirello I., Nicolini A., Meregaglia D., Burdick L. (2003). Humoral and cardiac effects of TIPS in cirrhotic patients with different “effective” blood volume. Hepatology.

[39] Gaba R.C., Parvinian A., Casadaban L.C., Couture P.M., Zivin S.P., Lakhoo J., Minocha J., Ray C.E., Knuttinen M.G., Bui J.T. (2015). Survival benefit of TIPS versus serial paracentesis in patients with refractory ascites: A single institution case-control propensity score analysis. Clin. Radiol..

[40] Ronald J., Rao R., Choi S.S., Kappus M., Martin J.G., Sag A.A., Pabon-Ramos W.M., Suhocki P.V., Smith T.P., Kim C.Y. (2019). No Increased Mortality After TIPS Compared with Serial Large Volume Paracenteses in Patients with Higher Model for End-Stage Liver Disease Score and Refractory Ascites. Cardiovasc. Interv. Radiol..

[41] Bischoff S.C., Bernal W., Dasarathy S., Merli M., Plank L.D., Schütz T., Plauth M. (2020). ESPEN practical guideline: Clinical nutrition in liver disease. Clin. Nutr..

[42] European Association for the Study of the Liver (2019). EASL Clinical Practice Guidelines on nutrition in chronic liver disease. J. Hepatol..

[43] Amodio P., Bemeur C., Butterworth R., Cordoba J., Kato A., Montagnese S., Uribe M., Vilstrup H., Morgan M.Y. (2013). The nutritional management of hepatic encephalopathy in patients with cirrhosis: International Society for Hepatic Encephalopathy and Nitrogen Metabolism Consensus. Hepatology.

[44] Merli M., Giusto M., Molfino A., Bonetto A., Rossi M., Ginanni Corradini S., Baccino F.M., Rossi Fanelli F., Costelli P., Muscaritoli M. (2013). MuRF-1 and p-GSK3β expression in muscle atrophy of cirrhosis. Liver Int..

[45] Kim G., Kang S.H., Kim M.Y., Baik S.K. (2017). Prognostic value of sarcopenia in patients with liver cirrhosis: A systematic review and meta-analysis. PLoS ONE.

[46] Lattanzi B., Nardelli S., Pigliacelli A., Di Cola S., Farcomeni A., D’Ambrosio D., Gioia S., Ginanni Corradini S., Lucidi C., Mennini G. (2019). The additive value of sarcopenia, myosteatosis and hepatic encephalopathy in the predictivity of model for end-stage liver disease. Dig. Liver Dis..

[47] Merli M., Giusto M., Giannelli V., Lucidi C., Riggio O. (2011). Nutritional status and liver transplantation. J. Clin. Exp. Hepatol..

[48] Mangana Del Rio T., Sacleux S.C., Vionnet J., Ichaï P., Denys A., Schneider A., Coilly A., Fraga M., Wetzel A., Koerfer J. (2023). Body composition and short-term mortality in patients critically ill with acute-on-chronic liver failure. JHEP Rep..

[49] Nardelli S., Lattanzi B., Torrisi S., Greco F., Farcomeni A., Gioia S., Merli M., Riggio O. (2017). Sarcopenia Is Risk Factor for Development of Hepatic Encephalopathy After Transjugular Intrahepatic Portosystemic Shunt Placement. Clin. Gastroenterol. Hepatol..

[50] Praktiknjo M., Clees C., Pigliacelli A., Fischer S., Jansen C., Lehmann J., Pohlmann A., Lattanzi B., Krabbe V.K., Strassburg C.P. (2019). Sarcopenia Is Associated with Development of Acute-on-Chronic Liver Failure in Decompensated Liver Cirrhosis Receiving Transjugular Intrahepatic Portosystemic Shunt. Clin. Transl. Gastroenterol..

[51] Gazda J., Di Cola S., Lapenna L., Khan S., Merli M. (2023). The Impact of Transjugular Intrahepatic Portosystemic Shunt on Nutrition in Liver Cirrhosis Patients: A Systematic Review. Nutrients.

[52] Montomoli J., Holland-Fischer P., Bianchi G., Grønbaek H., Vilstrup H., Marchesini G., Zoli M. (2010). Body composition changes after transjugular intrahepatic portosystemic shunt in patients with cirrhosis. World J. Gastroenterol..

[53] Bañares R., Albillos A., Nakum M., Gea S., Varghese A., Green W. (2023). An Economic Analysis of Transjugular Intrahepatic Portosystemic Covered Stent Shunt for Variceal Bleeding and Refractory Ascites in a Spanish Setting. Adv. Ther..

[54] Casadaban L.C., Parvinian A., Couture P.M., Minocha J., Knuttinen M.G., Bui J.T., Gaba R.C. (2014). Characterization of liver function parameter alterations after transjugular intrahepatic portosystemic shunt creation and association with early mortality. AJR Am. J. Roentgenol..

[55] Vizzutti F., Arena U., Rega L., Zipoli M., Abraldes J.G., Romanelli R.G., Tarquini R., Laffi G., Pinzani M. (2009). Liver failure complicating segmental hepatic ischaemia induced by a PTFE-coated TIPS stent. Gut.

[56] Cazzaniga M., Salerno F., Pagnozzi G., Dionigi E., Visentin S., Cirello I., Meregaglia D., Nicolini A. (2007). Diastolic dysfunction is associated with poor survival in patients with cirrhosis with transjugular intrahepatic portosystemic shunt. Gut.

[57] Billey C., Billet S., Robic M.A., Cognet T., Guillaume M., Vinel J.P., Péron J.M., Lairez o., Bureau C. (2019). A prospective study identifying predictive factors of cardiac decompensation after TIPS: The Toulouse algorithm. Hepatology.

[58] Riggio O., Angeloni S., Salvatori F.M., De Santis A., Cerini F., Farcomeni A., Attili A.F., Merli M. (2008). Incidence, natural history, and risk factors of hepatic encephalopathy after transjugular intrahepatic portosystemic shunt with polytetrafluoroethylene-covered stent grafts. Am. J. Gastroenterol..

[59] Riggio O., Nardelli S., Moscucci F., Pasquale C., Ridola L., Merli M. (2012). Hepatic encephalopathy after transjugular intrahepatic portosystemic shunt. Clin. Liver Dis..

[60] Bureau C., Thabut D., Jezequel C., Archambeaud I., D’Alteroche L., Dharancy S., Borentain P., Oberti F., Plessier A., De Ledinghen V. (2021). The Use of Rifaximin in the Prevention of Overt Hepatic Encephalopathy After Transjugular Intrahepatic Portosystemic Shunt: A Randomized Controlled Trial. Ann. Intern. Med..

[61] Nardelli S. Hepatic encephalopathy after transjugular intrahepatic portosystemic shunt does not increase mortality in patients with cirrhosis. Proceedings of the EASL Congress 2023.

[62] Unger L.W., Stork T., Bucsics T., Rasoul-Rockenschaub S., Staufer K., Trauner M., Maschke S., Pawloff M., Soliman T., Reiberger T. (2017). The role of TIPS in the management of liver transplant candidates. United Eur. Gastroenterol. J..

[63] Freeman R.B., FitzMaurice S.E., Greenfield A.E., Halin N., Haug C.E., Rohrer R.J. (1994). Is the transjugular intrahepatic portocaval shunt procedure beneficial for liver transplant recipients?. Transplantation.

[64] Menegaux F., Baker E., Keeffe E.B., Monge H., Egawa H., Esquivel C.O. (1994). Impact of transjugular intrahepatic portosystemic shunt on orthotopic liver transplantation. World J. Surg..

[65] Martin M., Zajko A.B., Orons P.D., Dodd G., Wright H., Colangelo J., Tartar R. (1993). Transjugular intrahepatic portosystemic shunt in the management of variceal bleeding: Indications and clinical results. Surgery.

[66] Woodle E.S., Darcy M., White H.M., Perdrizet G.A., Vesely T.M., Picus D., Hicks M., So S.K., Jendrisak M.D., McCullough C.S. (1993). Intrahepatic portosystemic vascular stents: A bridge to hepatic transplantation. Surgery.

[67] Moreno A., Meneu J.C., Moreno E., Fraile M., García I., Loinaz C., Abradelo M., Jiménez C., Gomez R., García-Sesma A. (2003). Liver transplantation and transjugular intrahepatic portosystemic shunt. Transplant. Proc..

[68] Dell’Era A., Grande L., Barros-Schelotto P., Turnes J., Fuster J., Charco R., García-Valdecasas J.C., Bosch J., García-Pagán J.C. (2005). Impact of prior portosystemic shunt procedures on outcome of liver transplantation. Surgery.

[69] Guerrini G.P., Pleguezuelo M., Maimone S., Calvaruso V., Xirouchakis E., Patch D., Rolando N., Davidson B., Rolles K., Burroughs A. (2009). Impact of tips preliver transplantation for the outcome posttransplantation. Am. J. Transplant..

[70] Barbier L., Hardwigsen J., Borentain P., Biance N., Daghfous A., Louis G., Botta-Fridlund D., Le Treut Y.P. (2014). Impact of transjugular intrahepatic portosystemic shunting on liver transplantation: 12-year single-center experience. Clin. Res. Hepatol. Gastroenterol..

[71] Fagiuoli S., Bruno R., Debernardi Venon W., Schepis F., Vizzutti F., Toniutto P., Senzolo M., Caraceni P., Salerno F., Angeli P. (2017). Consensus conference on TIPS management: Techniques, indications, contraindications. Dig. Liver Dis..

[72] Dariushnia S.R., Haskal Z.J., Midia M., Martin L.G., Walker T.G., Kalva S.P., Clark T.W., Ganguli S., Krishnamurthy V., Saiter C.K. (2016). Quality Improvement Guidelines for Transjugular Intrahepatic Portosystemic Shunts. J. Vasc. Interv. Radiol..

[73] Sellers C.M., Nezami N., Schilsky M.L., Kim H.S. (2019). Transjugular intrahepatic portosystemic shunt as a bridge to liver transplant: Current state and future directions. Transplant. Rev..

[74] Balducci D., Montori M., De Blasio F., Di Bucchianico A., Argenziano M.E., Baroni G.S., Scarpellini E. (2023). The Role of Transjugular Intrahepatic Portosystemic Shunt (TIPS) in Treating Portal Hypertension in Patients with Hepatocellular Carcinoma. Medicina.

[75] Chen Z.X., Qiu Z.K., Wang G.B., Wang G.S., Jiang W.W., Gao F. (2023). Safety and effectiveness of transjugular intrahepatic portosystemic shunt in hepatocellular carcinoma patients with portal hypertension: A systematic review and meta-analysis. Clin. Radiol..

[76] Bjørneboe M., Andersen J.R., Christensen U., Skinhøj P., Jensen O.M. (1985). Does a portal-systemic shunt increase the risk of primary hepatic carcinoma in cirrhosis of the liver?. Scand J. Gastroenterol..

[77] Bañares R., Núñez O., Escudero M., Fernández C., Vaquero J., Beceiro I., Echenagusía A., Clemente G., Santos L. (2005). Patients with cirrhosis and bare-stent TIPS may have increased risk of hepatocellular carcinoma. Hepatology.

[78] Chen B., Pang L., Chen H.B., Wu D.B., Wang Y.H., Chen E.Q. (2019). TIPS Is Not Associated with a Higher Risk of Developing HCC in Cirrhotic Patients: A Systematic Review and Meta-analysis. J. Clin. Transl. Hepatol..

[79] Kang J.W., Kim J.H., Ko G.Y., Gwon DIl Yoon H.K., Sung K.B. (2012). Transarterial chemoembolization for hepatocellular carcinoma after transjugular intrahepatic portosystemic shunt. Acta Radiol..

[80] Tesdal I.K., Wikström M., Flechtenmacher C., Filser T., Dueber C. (2006). Percutaneous treatment of hepatocellular carcinoma in patients with transjugular intrahepatic portosystemic shunts. Cardiovasc. Interv. Radiol..

[81] Wang Z., Zhang H., Zhao H., Wang X., Tsauo J., Luo X., Li X. (2014). Repeated transcatheter arterial chemoembolization is safe for hepatocellular carcinoma in cirrhotic patients with transjugular intrahepatic portosystemic shunt. Diagn. Interv. Radiol..

[82] Donahue L.A., Kulik L., Baker T., Ganger D.R., Gupta R., Memon K., Abecassis M.M., Salem R., Lewandowski R.J. (2013). Yttrium-90 radioembolization for the treatment of unresectable hepatocellular carcinoma in patients with transjugular intrahepatic portosystemic shunts. J. Vasc. Interv. Radiol..

[83] Park J.K., Al-Tariq Q.Z., Zaw T.M., Raman S.S., Lu D.S. (2015). Radiofrequency Ablation for the Treatment of Hepatocellular Carcinoma in Patients with Transjugular Intrahepatic Portosystemic Shunts. Cardiovasc. Interv. Radiol..

[84] Kohi M.P., Fidelman N., Naeger D.M., LaBerge J.M., Gordon R.L., Kerlan R.K. (2013). Hepatotoxicity after transarterial chemoembolization and transjugular intrahepatic portosystemic shunt: Do two rights make a wrong?. J. Vasc. Interv. Radiol..

[85] Kuo Y.C., Kohi M.P., Naeger D.M., Tong R.T., Kolli K.P., Taylor A.G., Laberge J.M., Kerlan R.K., Fidelman N. (2013). Efficacy of TACE in TIPS patients: Comparison of treatment response to chemoembolization for hepatocellular carcinoma in patients with and without a transjugular intrahepatic portosystemic shunt. Cardiovasc. Interv. Radiol..

[86] Moreau R., Jalan R., Gines P., Pavesi M., Angeli P., Cordoba J., Durand F., Gustot T., Saliba F., Domenicali M. (2013). Acute-on-chronic liver failure is a distinct syndrome that develops in patients with acute decompensation of cirrhosis. Gastroenterology.

[87] European Association for the Study of the Liver (2018). EASL Clinical Practice Guidelines for the management of patients with decompensated cirrhosis. J. Hepatol..

[88] Gu W., Kimmann M., Laleman W., Praktiknjo M., Trebicka J. (2023). To TIPS or Not to TIPS in High Risk of Variceal Rebleeding and Acute-on-Chronic Liver Failure. Semin. Liver Dis..

[89] Wong F., Pantea L., Sniderman K. (2004). Midodrine, octreotide, albumin, and TIPS in selected patients with cirrhosis and type 1 hepatorenal syndrome. Hepatology.

[90] Testino G., Leone S., Ferro C., Borro P. (2012). Severe acute alcoholic hepatitis and hepatorenal syndrome: Role of transjugular intrahepatic portosystemic stent shunt. J. Med. Life.

[91] Brensing K.A., Textor J., Perz J., Schiedermaier P., Raab P., Strunk H., Klehr H.U., Kramer H.J., Spengler U., Schild H. (2000). Long term outcome after transjugular intrahepatic portosystemic stent-shunt in non-transplant cirrhotics with hepatorenal syndrome: A phase II study. Gut.

[92] Song T., Rössle M., He F., Liu F., Guo X., Qi X. (2018). Transjugular intrahepatic portosystemic shunt for hepatorenal syndrome: A systematic review and meta-analysis. Dig. Liver Dis..

[93] Ripoll C., Platzer S., Franken P., Aschenbach R., Wienke A., Schuhmacher U., Teichgräber U., Stallmach A., Steighardt J., Zipprich A. (2023). Liver-HERO: Hepatorenal syndrome-acute kidney injury (HRS-AKI) treatment with transjugular intrahepatic portosystemic shunt in patients with cirrhosis-a randomized controlled trial. Trials.

[94] Premkumar M., Devurgowda D., Vyas T., Shasthry S.M., Khumuckham J.S., Goyal R., Thomas S.S., Kumar G. (2019). Left Ventricular Diastolic Dysfunction is Associated with Renal Dysfunction, Poor Survival and Low Health Related Quality of Life in Cirrhosis. J. Clin. Exp. Hepatol..

[95] Merli M., Valeriano V., Funaro S., Attili A.F., Masini A., Efrati C., De C.S., Riggio O. (2002). Modifications of cardiac function in cirrhotic patients treated with transjugular intrahepatic portosystemic shunt (TIPS). Am. J. Gastroenterol..

[96] Radunski U.K., Kluwe J., Klein M., Galante A., Lund G.K., Sinning C., Bohnen S., Tahir E., Starekova J., Bannas P. (2021). Cardiovascular magnetic resonance demonstrates structural cardiac changes following transjugular intrahepatic portosystemic shunt. Sci. Rep..

[97] Filì D., Falletta C., Luca A., Hernandez Baravoglia C., Clemenza F., Miraglia R., Scardulla C., Tuzzolino F., Vizzini G., Gridelli B. (2015). Circulatory response to volume expansion and transjugular intrahepatic portosystemic shunt in refractory ascites: Relationship with diastolic dysfunction. Dig. Liver Dis..

[98] Meucci M.C., Hoogerduijn Strating M.M., Butcher S.C., van Rijswijk C.S.P., Van Hoek B., Delgado V., Bax J.J., Tushuizen M.E., Marsan N.A. (2022). Left atrial dysfunction is an independent predictor of mortality in patients with cirrhosis treated by transjugular intrahepatic portosystemic shunt. Hepatol. Commun..

[99] Rabie R.N., Cazzaniga M., Salerno F., Wong F. (2009). The use of E/A ratio as a predictor of outcome in cirrhotic patients treated with transjugular intrahepatic portosystemic shunt. Am. J. Gastroenterol..

